# Supplementary material for: BASALT refines binning from metagenomic data and increases resolution of genome-resolved metagenomic analysis
Source: Nat Commun. 2024 Mar 11;15:2179. doi: 10.1038/s41467-024-46539-7 (PMC10928208; doi:10.1038/s41467-024-46539-7)
Supplement: Supplementary file 1 — Supplementary Information [file 41467_2024_46539_MOESM1_ESM.pdf]

BASALT refines binning from metagenomic data and increases resolution of genome-resolved metagenomic analysis

Supplementary Note 1

BASALT improves recognition of non-redundant bins.

BASALT platform recognizes and accepts various types of metagenomic sequence data as initial input files, including short read sequences (SRS), long read sequences (LRS) and hybrid sequences to be used in different assembly strategies. The advantage of input with multiple files is not limited to the reduction of computational time (*i.e.,* process all datasets with binning together other than individually) but could also generate more bins than individually assembled samples. For example, using SRS, binning using multiplexed samples generated 16.3%, 14.2% and 11.1% more non-redundant MAGs than single-assembled samples when using DASTool (MCM), VAMB and metaWRAP (MCM), respectively on CAMI-medium dataset (Supplementary Figure 4, Supplementary Data 3). Such increasing rate on CAMI-high dataset were 8.2%, 11.0% and 9.0%, respectively (Supplementary Figure 3, Supplementary Data 3).

Despite the advantage above, a major drawback using multiplexed samples was the generation of replicated bins (or pseudo-genomes), which is wasteful of computing power. To address this issue, the Bin Selection Module includes a bin dereplication function to facilitate identification of redundant bins generated by co-assembled contigs in SRS data [1]. After processing with the BASALT Bin Selection module, no redundant bins were found among bin sets of CAMI-medium or CAMI-high datasets assembled with SPAdes, whereas VAMB, DASTool, and metaWRAP, respectively generated 77.1%, 85.9%, and 95.0% redundant bins in CAMI-medium reads data, and 52.3%, 84.8%, and 72.7% redundant MAGs in the CAMI-high dataset (Supplementary Figure 3D, 4D, Supplementary Data 3). On the other hand, no redundancy was found in BASALT produced MAGs. In comparison of MAGs (Quality score ≥ 50) obtained via different toolkits, BASALT resulted in 27.1%, 7.0%, 1.7% (CAMI-

medium) and 50.9%, 50%, 11.7% (CAMI-high) more non-redundant MAGs than DASTool, VAMB and metaWRAP, respectively (Supplementary Figure 4D, Supplementary Data 3). Moreover, in top-qualitied CAMI-high MAGs (Quality score ≥ 90), BASALT obtained 38%, 47.7% and 17.6% more non-redundant MAGs than DASTool, VAMB and metaWRAP, respectively (Supplementary Figure 3D, Supplementary Data 3). MAGs recovered from SPAdes assembled contigs on CAMI-medium dataset, as well as MAGs recovered from MEGAHIT assembled contigs on CAMI-medium and -high datasets showed similar trends and significance in comparison between BASALT and other tools (Supplementary Data 3). These results indicated that BASALT could be used to eliminate redundant bins from data processed by other tools.

Supplementary Note 2

BASALT optimizes bins from lower-performance options and other pipelines.

In BASALT, each module can be applied independently, consequently enabling refinement of user binsets with optimal parameters appropriate with their specific data, and improving the quality of bins acquired from other tools, especially existing binsets or assemblies of publicly available data. In addition to eliminating bin redundancy in co-assembled datasets, post-binning refinement modules (*i.e.,* Bin Selection, Refinement, and Gap Filling Modules) can also remove redundant bins generated by other tools to improve bin Quality. In CAMI-high binsets assembled by SPAdes with VAMB, DASTool, or metaWRAP, refinement with BASALT respectively increased the number of high-quality MAGs (Quality ≥ 80) by 12.6%, 11.7%, and 13.0% (Supplementary Figure 6A & B). In MEGAHIT-assembled datasets, the number of high-quality MAGs were increased by 17.0%, 31.4%, and 18.0% compared to VAMB, DASTool, and metaWRAP outputs, respectively (Supplementary Figure 6C & D). Moreover, BASALT can also directly integrate LRS assemblies with the Gap Filling module to elongate contigs and fill gaps in bins without conducting hybrid assembly (Supplementary Figure 6, Supplementary Data 3), which can also improve bin Quality and continuity. This improvement resulted

in 11.2%, 11.2% and 12.4% more top-qualitied (Quality score ≥ 80) and non-redundant MAGs retrieved using SPAdes, and 9.0%, 25.0% and 10.1% using MEGAHIT via VAMB, DASTool, and metaWRAP, respectively on CAMI-high dataset (Supplementary Data 3). Less improvement was found on CAMI-medium dataset (Supplementary Data 3), suggested that BASALT with better performance on datasets with higher complexity. Overall, BASALT could be integrated into other binning tools for higher MAG number and qualities, especially in the presence of long sequence reads to largely augment MAG integrity and qualities.

Supplementary Note 3

Better performance of BASALT than metaWRAP and MAG-HiFi pipeline on real samples.

To enhance the assessment the performance of BASALT across diverse sample types in addition to saline lake sediment samples, we further compared the metagenome-assembled genomes (MAGs) produced by BASALT against those generated by metaWRAP from same assemblies from nine sample sources. These samples included marine, human gut (Dataset 1), activated sludge, and Antarctic soil, where both short-read sequences (SRS) and long-read sequences (LRS) are available. Moreover, we also compared BASALT-generated MAGs with those from the MAG-HiFi pipeline (v2.1.0, https://github.com/PacificBiosciences/pb-metagenomics-tools) using identical assemblies from samples sequenced with PacBio HiFi, including human gut (Dataset 2, a different study from Dataset 1), chicken gut, sheep gut, hot spring sediments, and anerobic digesters.

Results showed that in SRS+LRS datasets, BASALT consistently outperformed metaWRAP by generating 9-42% more MAGs. In PacBio HiFi datasets, BASALT obtained 7-28% more MAGs compared to MAG-HiFi pipeline (Supplementary Figure 7, Supplementary Data 10). This was particularly conspicuous in high-quality MAGs, where BASALT retrieved 26-96% and 18-46% more MAGs than metaWRAP and the MAG-HiFi pipeline, respectively (Supplementary Figure 7,

Supplementary Data 10). A quality assessment revealed that MAGs obtained by BASALT had significantly better quality than the same MAGs acquired via metaWRAP (ANI $\geq$ 99%, AF $\geq$ 60%, hereafter shared MAGs) across all sample types except the Antarctic soil sample (one-way ANOVA, $P < 0.01$, Supplementary Figure 8A). The above data in conjunction with the results from Aiding Lake samples suggested that BASALT is capable of recovering more MAGs from different types of datasets.

Furthermore, owing to the implementation of LRS, we evaluated the N50 value of shared MAGs generated by both BASALT and metaWRAP, as well as between BASALT and the MAG-HiFi pipeline. Results showed that MAGs from BASALT yielded in a higher N50 value from SRS+LRS samples compared to metaWRAP (Supplementary Figure 8B), while merely no difference was found from HiFi samples when compared to MAGs obtained by MAG-HiFi pipeline (Supplementary Figure 8C), suggesting that BASALT has better performance of obtaining longer contigs in these SRS+LRS samples.

To further assess the MAGs from marine and human gut samples, we compared the average coverage of each MAG across these sample types. Results showed that MAGs uniquely derived from BASALT had a lower average coverage than that of metaWRAP in both marine and human gut samples (Supplementary Figure 9A). Particularly, the difference was statistically significant in marine samples (one-way ANOVA, $P < 0.05$). An ORF annotation revealed that BASALT MAGs contained more ORF than metaWRAP, regardless of the MAGs being classified or unclassified against NCBI RefSeq database (Supplementary Figure 9B). In conclusion, the results suggested that BASALT outperformed metaWRAP in generating more and better-quality MAGs across various sample types.

Supplementary Methods

Architecture of neural networks.

To identify the redundant bins produced by Automated Binning Module from co-assembly datasets, a

total of 20 neural networks were ensembled, each of them consists of multiple Fully-Connected (FC) layers trained on the data with different hyper-parameters. each neural network has three blocks with multiple Feed-Forward Network (FFN) modules in first two block and one FC classifier to classify 'redundant' or 'non-redundant' in the last block. The FFN module comprises two FC layers as two linear transformations, each with a ReLU as activation function and followed by a Batch Normalization (BN) [2]. The FFN value is calculated by Supplementary Equation (1):

Supplementary Equation (1)

$$FFN(x) = BN(\max(0, x\, W_1 + b_1)\, W_2 + b_2)\,,$$

in which x is the data feature, W_1 and W_2 are parameters of the first and second layer, respectively, while b_1 and b_2 represent the biases of the two FC layers, respectively. BN assists to normalize the data distribution in each layer, which is formulated as Supplementary Equation (2):

Supplementary Equation (2)

$$BN(x) = \gamma \frac{x - E[x]}{\sqrt{Var[x] + \epsilon}} + \beta\,,$$

where $E[x]$ and $Var[x]$ are the mean and variances of input feature, γ and β are learnable parameters to finetune the data distribution. ϵ is infinitesimal for stable division operations.

To make each network can be trained deeply, we also apply residual connection [3] in the first two blocks, with the whole computation process in the neural networks as Supplementary Equation (3):

Supplementary Equation (3)

$$x' = x + FFN(x + FFN(x)),$$

$$y = x' W_{fc} + b_{fc},$$

where W_{fc} and b_{fc} are the parameters of final classifier layer.

In addition, we also apply feature engineering on the input dataset. In the training stage, the labelled data was split as training sets and testing sets, with the mean and maximized value of the data

normalized in each input dataset. Then, the original input data with 10 dimensions was concatenated with 15 additional feature dimensions in the time frequency domain, including means, variance, square root amplitude, margin index, etc. Finally, the 40-dimension data features were generated as the final input data x for neural networks.

Loss function

As we formulate this problem as a binary classification problem, cross entropy loss was directly used in training, formulated as Supplementary Equation (4):

Supplementary Equation (4)

$$loss = -\sum_{c=1}^{C} w_c \log \frac{\exp(x_c)}{\sum_i^C \exp(x_i)} y_c,$$

where x was used as the input, x as the target, and w represents the weight, C represents the number of classes. Here, we set $C = 2$ in the binary classification problem. The Formula S4 as cross entropy loss was used to train each neural network.

Implementations

Neural networks were trained using batch size 1024 with 1 NVIDIA 3090 GPUs. Pytorch [4] library was adopted to implement models and conduct all experiments. These models were trained for 100 epochs using AdamW [5] as the optimizer and cosine learning rate decay.

Supplementary Figures

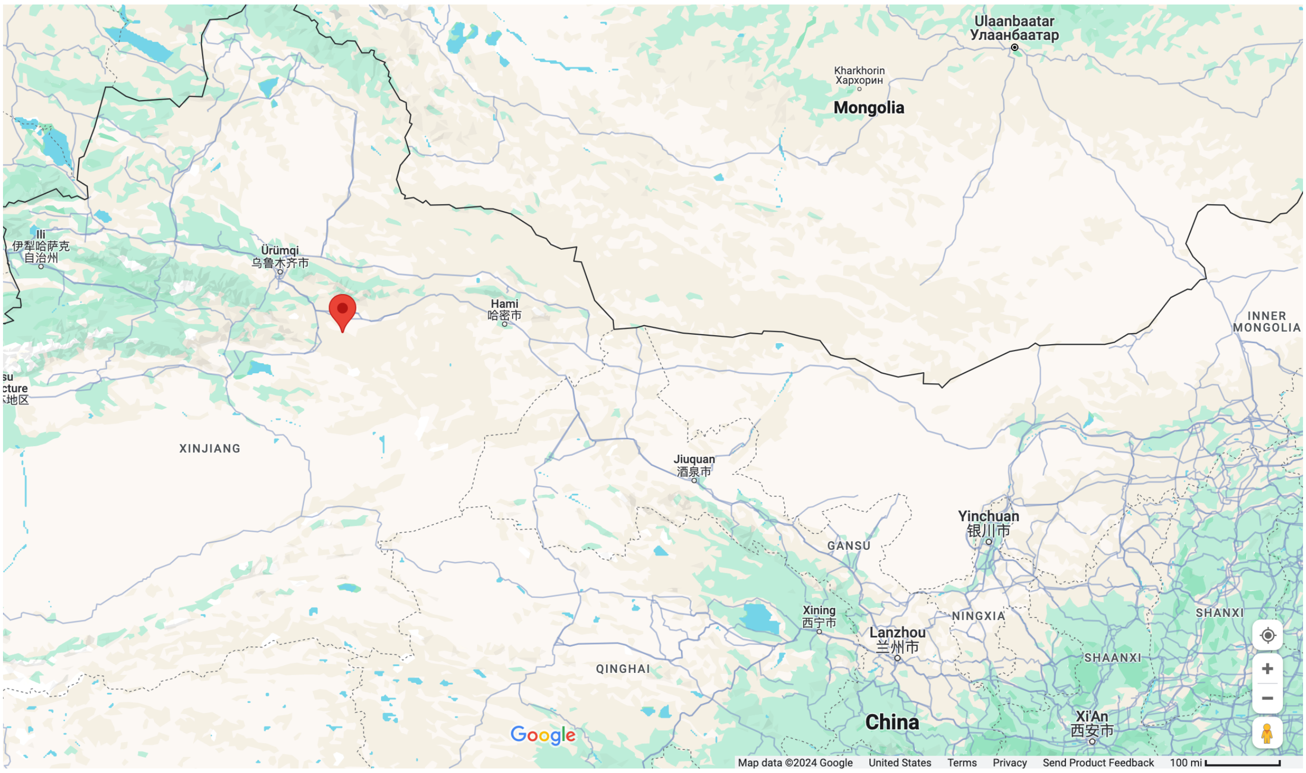

Supplementary Figure 1. Location of Aiding Lake indicated with the red pin on the map. The map was generated by Google Map (Map data ©2024 Google).

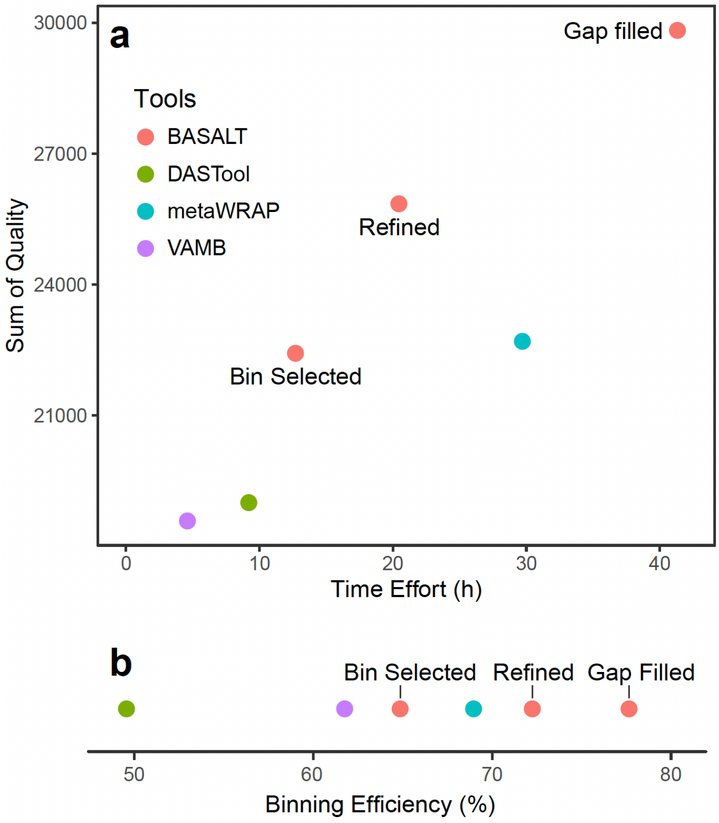

Supplementary Figure 2. Evaluation of Binning Efficiency on Opera-MS assembled CAMI-high dataset. a) Computation time of Binning based on CAMI-high hybrid assemblies using BASALT (red), DASTool (green), metaWRAP (cyan) and VAMB (purple). The x axis indicates time effort (hours), and y axis indicates summed Quality (Completeness – 5*Contamination) of filtered bins (completeness ≥ 35 and contamination < 20). BASALT results were showed finishing Bin Selection, Refinement, and Gap Filling Modules, respectively. b) Reads usage efficiency of BASALT (red), DASTool (green), metaWRAP (cyan) and VAMB (purple).

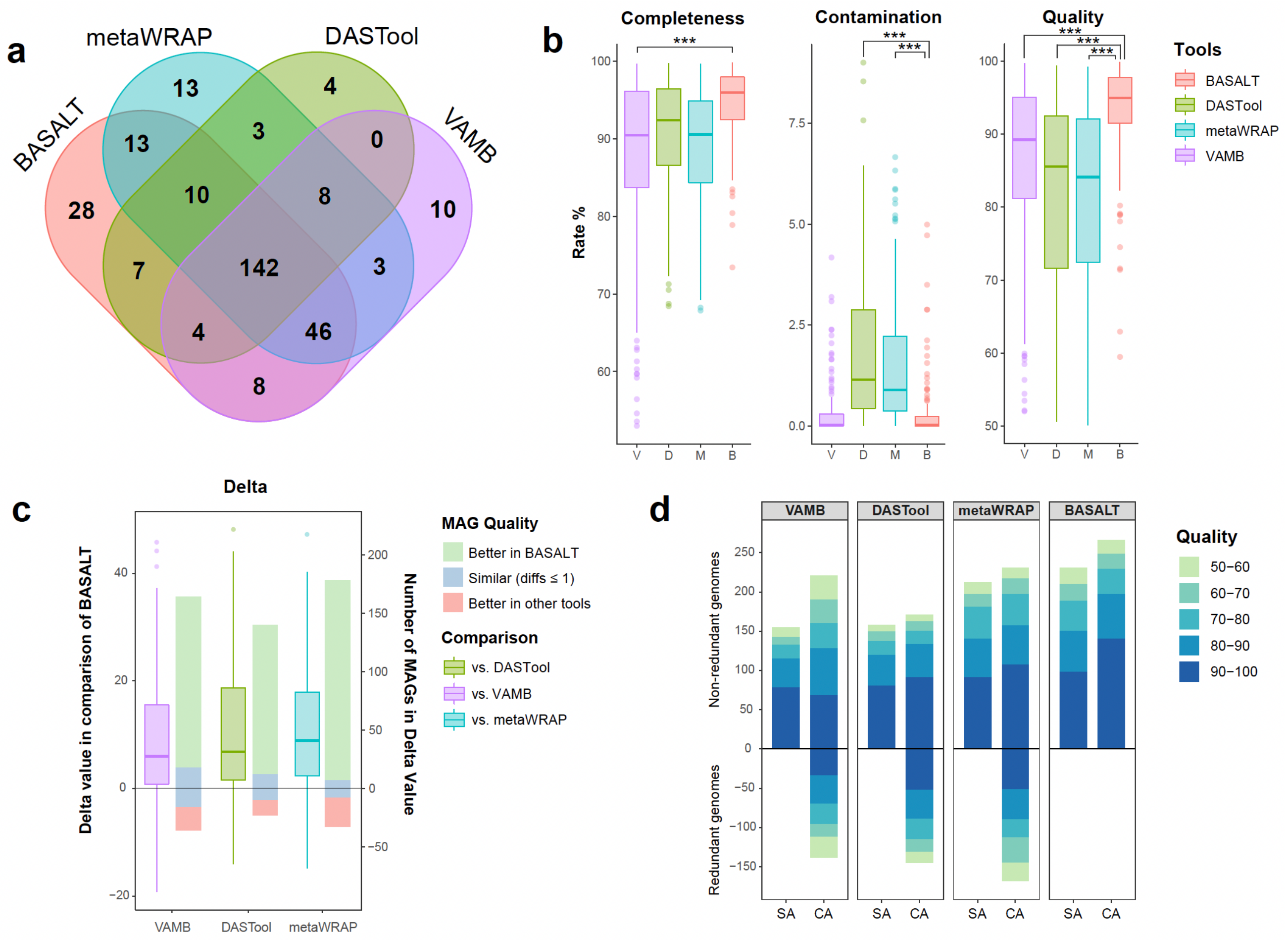

Supplementary Figure 3. Comparison of BASALT with other binning tools/pipelines on SPAdes assembled CAMI-high dataset. a) Venn diagram showing number of MAGs recovered using different tools. There were 142 MAGs found shared across all tools, while 28, 13, 4 and 10 MAGs were uniquely recovered using BASALT (red), metaWRAP (cyan), DASTool (green) and VAMB (purple) pipelines, respectively. b) Completeness, Contamination, and Quality of 142 shared MAGs recovered using VAMB (V, purple), DASTool (D, green), metaWRAP (M, cyan), and BASALT (B, red). MAGs recovered using BASALT had higher completeness and lower contamination, resulting in significant higher Quality value compared to VAMB, DASTool, and metaWRAP (Tukey test, Benjamini–Hochberg adjusted $P < 1 \times 10^{-7}$). The boxplot shows the distribution of data, the central dot in the box represents the median, the box bounds represent the 25th and 75th percentiles, and

whiskers represent the minima to maxima values. c) Pairwise comparison of MAGs shared by VAMB (purple, 200 MAGs), DASTool (green, 163 MAGs), and metaWRAP (cyan, 211 MAGs), respectively. Overall, BASALT was superior in obtaining higher qualitied MAGs compared to other tools. The number of MAGs that BASALT gained higher quality value (bars in light green) was much more than the number of MAGs that other tools gained higher quality value (bars in light red) or had similar quality value (difference of value ≤ 1, bars in light blue). The boxplot shows the distribution of data, the central dot in the box represents the median, the box bounds represent the 25th and 75th percentiles, and whiskers represent the minima to maxima values. d) Number of MAGs recovered from CAMI-high dataset using DASTool, VAMB, metaWRAP and BASALT. In the first three tools, Co-assembly (CA) resulted in higher number of non-redundant MAGs compare to single assembly (SA) approach, while BASALT generated the highest quality and number of MAGs compared to other approaches. Color of bars indicated the quality of MAGs (50-100, from light to dark).

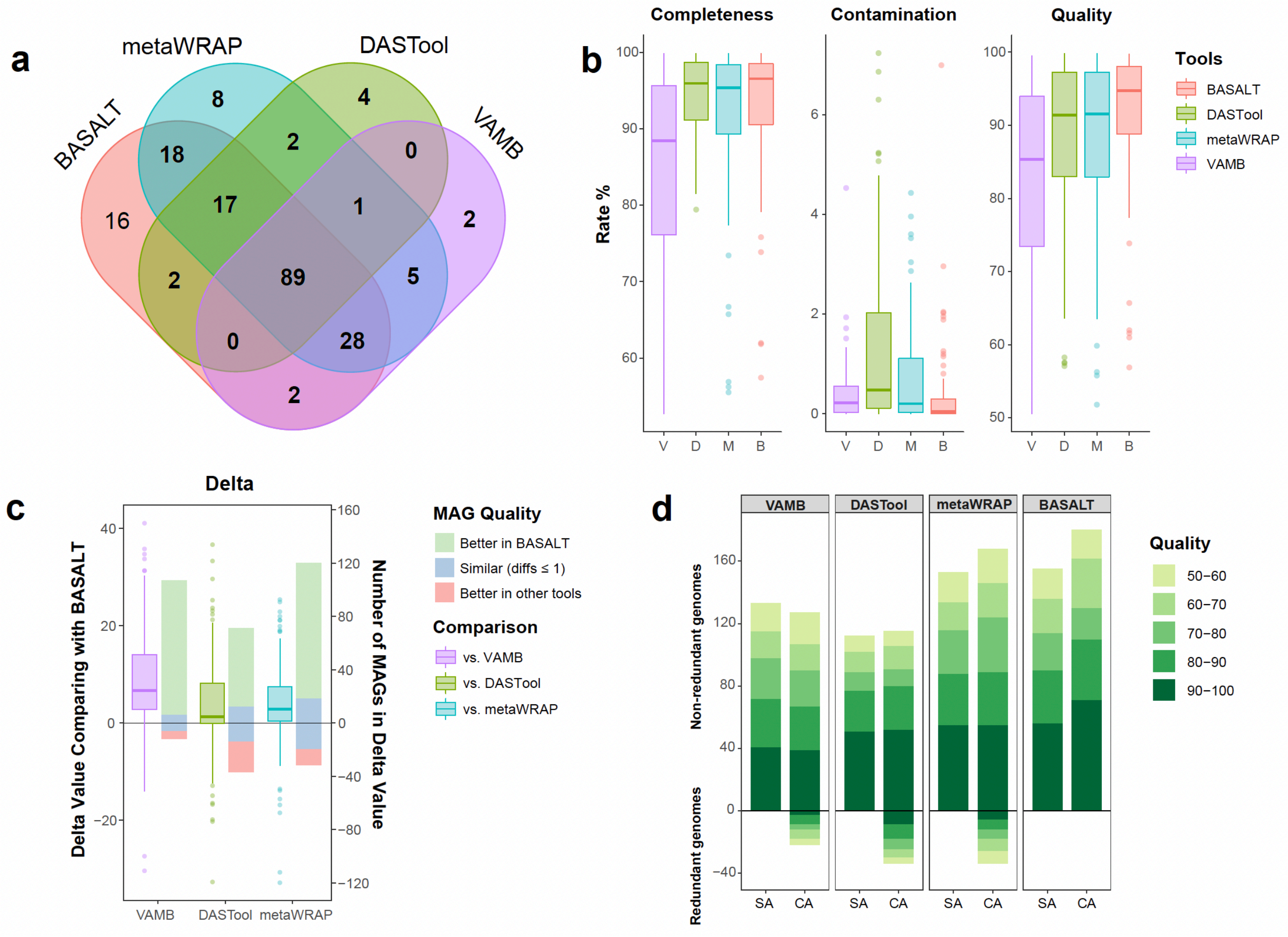

Supplementary Figure 4. Comparison of BASALT with other binning tools/pipelines on MEGAHIT assembled CAMI-high dataset. a) Venn diagram showing number of MAGs recovered using different tools. There were 89 MAGs found shared across all tools, while 16, 8, 4 and 2 MAGs were uniquely recovered using BASALT (red), metaWRAP (cyan), DASTool (green) and VAMB (purple) pipelines, respectively. b) Completeness, Contamination, and Quality of 89 shared MAGs recovered using VAMB (V, purple), DASTool (D, green),

metaWRAP (M, cyan), and BASALT (B, red). MAGs recovered using BASALT had higher completeness and lower contamination, resulting in higher Quality value compared to VAMB, DASTool, and metaWRAP. The boxplot shows the distribution of data, the central dot in the box represents the median, the box bounds represent the 25th and 75th percentiles, and whiskers represent the minima to maxima values. c) Pairwise comparison of MAGs shared by VAMB (purple, 119 MAGs), DASTool (green, 108 MAGs), and metaWRAP (cyan, 152 MAGs), respectively. Overall, BASALT was superior in obtaining higher qualitied MAGs compared to other tools. The number of MAGs that BASALT gained higher quality value (bars in light green) was much more than the number of MAGs that other tools gained higher quality value (bars in light red) or had similar quality value (difference of value ≤ 1, bars in light blue). The boxplot shows the distribution of data, the central dot in the box represents the median, the box bounds represent the 25th and 75th percentiles, and whiskers represent the minima to maxima values. d) Number of MAGs recovered from CAMI-high dataset using DASTool, VAMB, metaWRAP and BASALT. In the first three tools, Co-assembly (CA) resulted in higher number of non-redundant MAGs compare to single assembly (SA) approach, while BASALT generated the highest quality and number of MAGs compared to other approaches. Color of bars indicated the quality of MAGs (50-100, from light to dark).

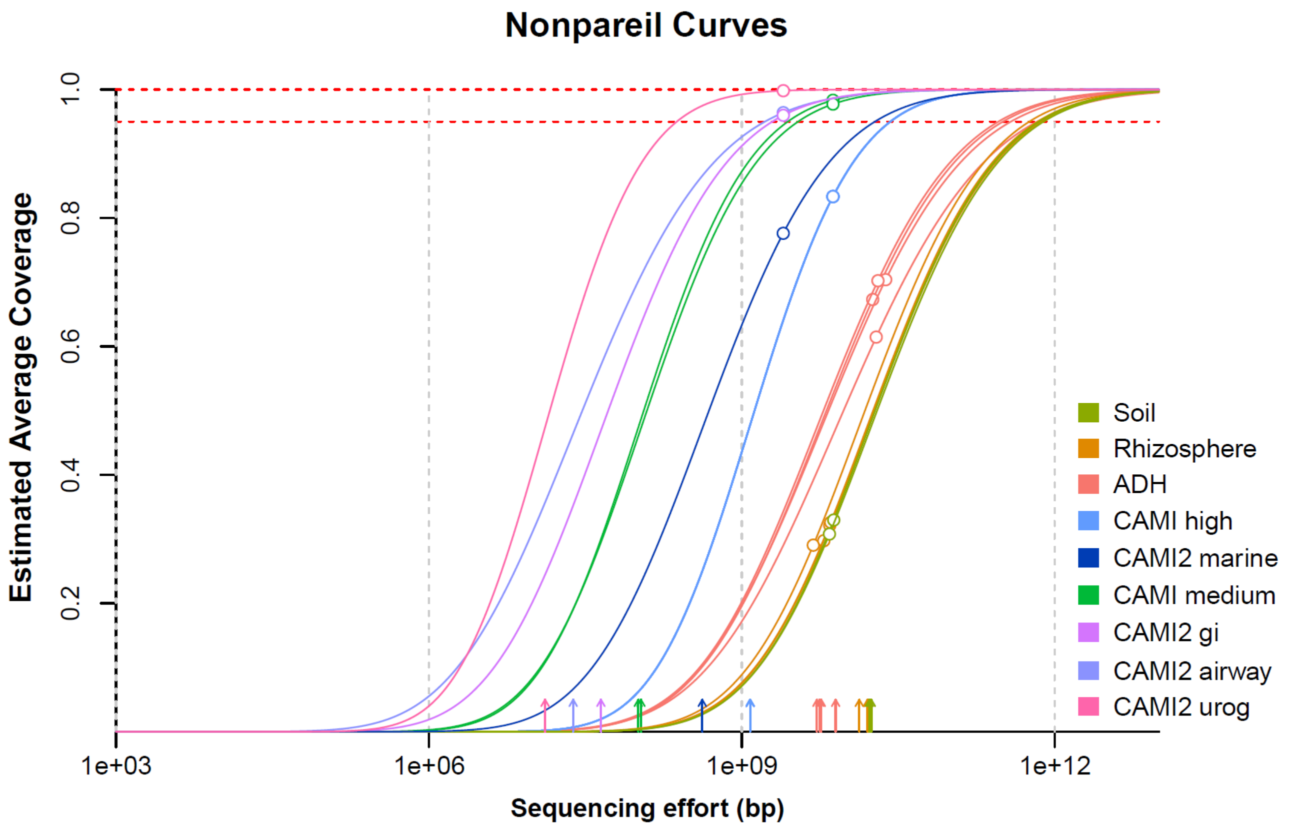

Supplementary Figure 5. Estimation of microbial diversity on CAMI medium (green), CAMI high (blue), CAMI II challenge (navy – marine, violet – gi, purple – airway, pink – urog), Aiding Lake sediment, soil and rhizosphere samples using Nonpareil. Top broken lines indicate 95% and 100% sequence coverage, respectively,

hallowed circles on the curves indicate the actual sample size, and arrows at the bottom indicate the sequencing effort at 50% sequence coverage.

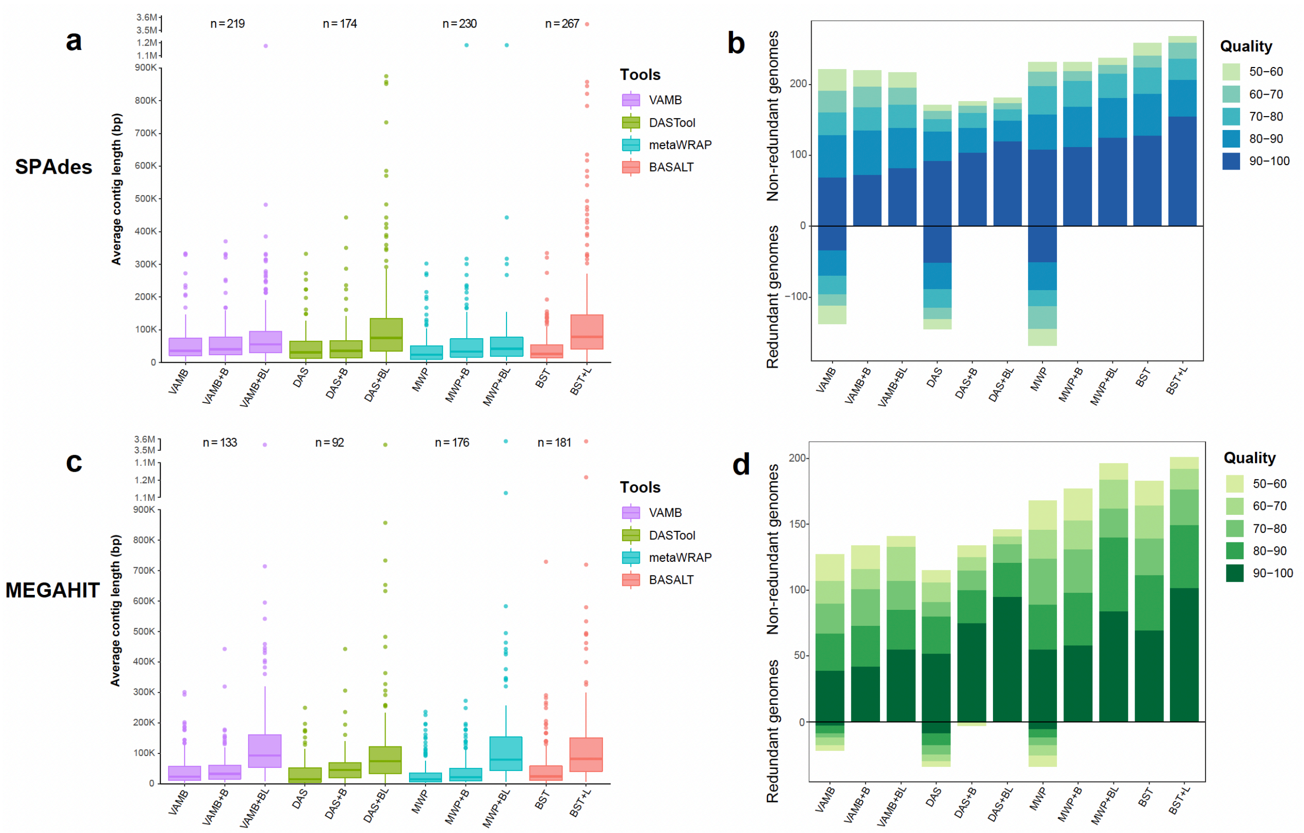

Supplementary Figure 6. Evaluation of BASALT refinement modules of genomes recovered by other binning tools/pipelines on CAMI-high dataset. Average length per contig of MAGs obtained via VAMB (purple), DASTool (DAS, green), metaWRAP (MWP, cyan) and BASALT (BST, red) were assessed based on a) SPAdes and c) MEGAHIT assemblers before (DAS, VAMB and MWP) and after BASALT refinement using short reads only (DAS+B, VAMB+B, MWP +B and BST) or combined short/long reads (DAS+BL, VAMB+BL, MWP+BL and BST+L). The sample size was shown on top of each tool group. The boxplot shows the distribution of data, the central dot in the box represents the median, the box bounds represent the 25th and 75th percentiles, and whiskers represent the minima to maxima values. Number of non-redundant and redundant MAGs obtained via different tools were summarized based on b) SPAdes and d) MEGAHIT assemblers before (DAS, VAMB and MWP) and after (DAS+B, DAS+BL, VAMB+B, VAMB+BL, MWP +B, MWP+BL, BST and BST+L) BASALT refinement. Color of bars indicated the quality of MAGs (50-100, from light to dark).

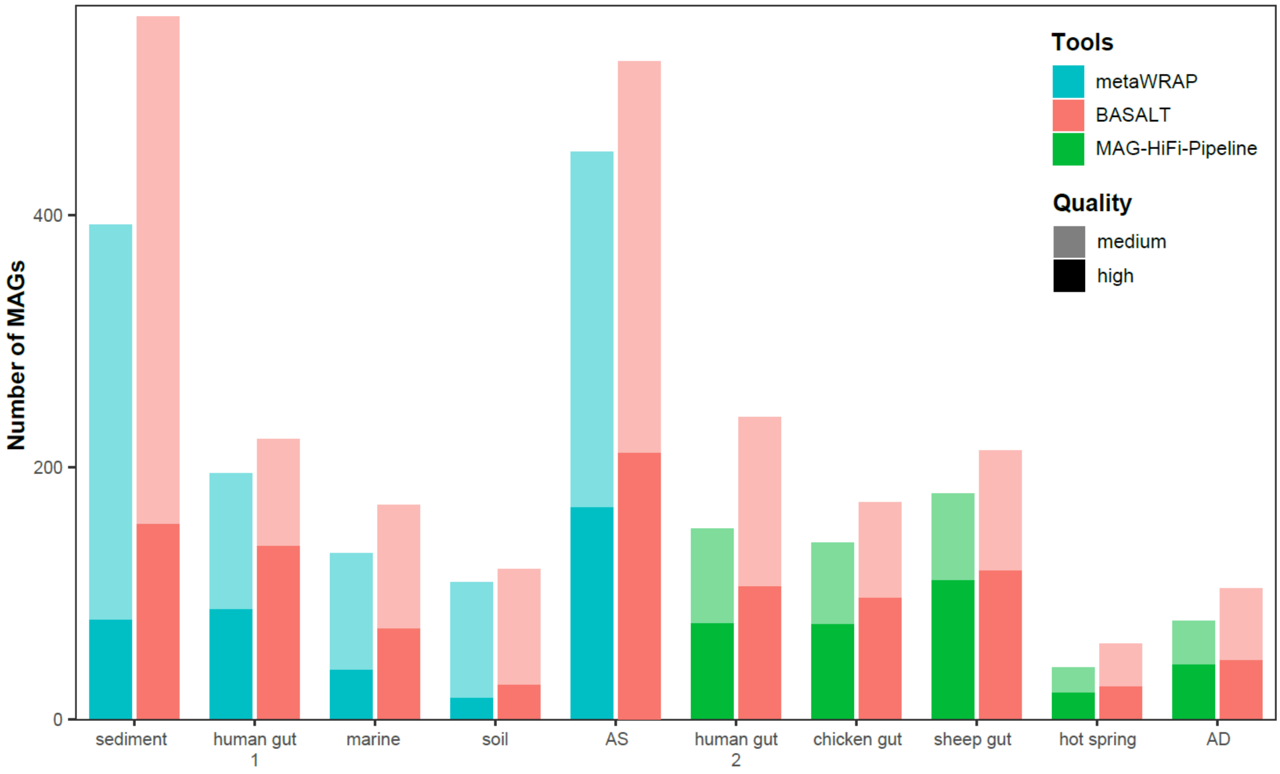

Supplementary Figure 7. Comparison of MAGs obtained by BASALT and metaWRAP on metagenomic datasets from lake sediments, human gut (Dataset 1, SRS+LRS), marine water, Antarctic soil, activated sludge (AS), human gut (Dataset 2, PacBio HiFi LRS), chicken gut, hot spring sediments, and anerobic digesters (AD). Colors of bars indicate tools (cyan: metaWRAP, red: BASALT, green: MAG-HiFi pipeline) used to retrieve MAGs. Light colors indicate medium-quality MAGs (completeness ≥ 50% and ≤ 90 %, contamination ≥ 5% and ≤ 10 %), and dark colors indicate high-quality MAGs (completeness ≥ 90 %, contamination ≤ 5 %). There were more BASALT MAGs recovered across various datasets than other tools, especially in high-quality MAGs.

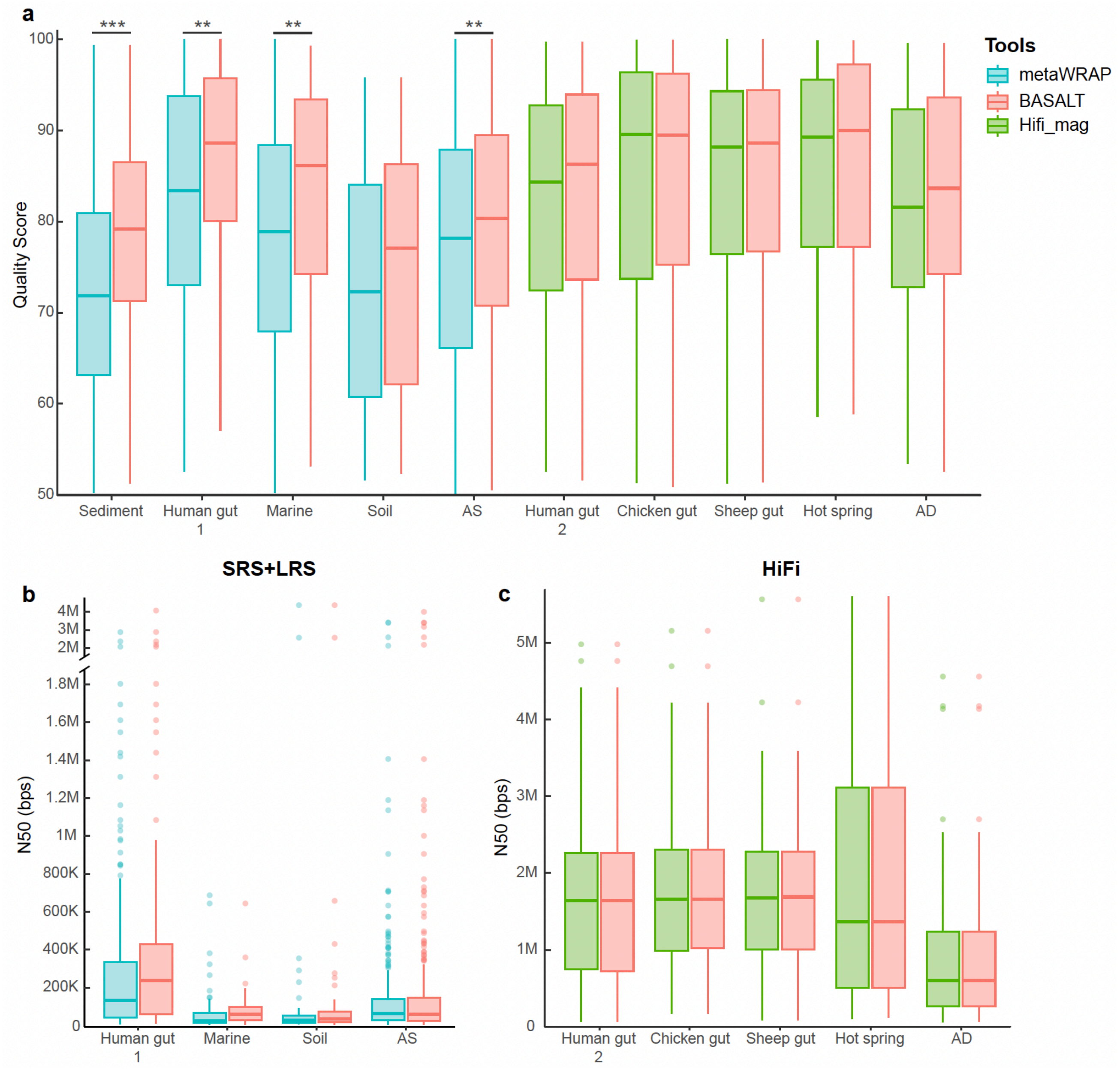

Supplementary Figure 8. Comparison of shared MAGs (ANI ≥ 99%, AF ≥ 60%) obtained by BASALT, metaWRAP, and MAG-HiFi pipeline on metagenomic dataset of lake sediments, human gut (Dataset 1, SRS+LRS, n = 140), marine water (n = 77), Antarctic soil (n = 58), activated sludge (AS, n = 338), human gut (Dataset 2, PacBio HiFi LRS, n = 119), chicken gut (n = 107), sheep gut (n = 166), hot spring sediments (n = 34), and anerobic digesters (AD, n = 69) real samples. a) MAG qualities of ten real samples. BASALT MAGs (red) had significant higher quality score ($P < 0.05$) than metaWRAP MAGs (cyan) in lake sediment (one-way ANOVA, $P = 1.76 \times 10^{-12}$), human gut Dataset 1 (one-way ANOVA, $P = 0.0026$), marine (one-way ANOVA, $P = 0.0053$), and AS (one-way ANOVA, $P = 0.0039$) samples. b) N50 value of shared MAGs retrieved by metaWRAP (cyan) and BASALT (red) from human gut Dataset 1, marine, Antarctic soil, and AS samples. Shared MAGs retrieved by BASALT had slightly higher N50 value than metaWRAP across all SRS+LRS samples except AS sample. c) N50 value of shared MAGs retrieved by MAG-HiFi pipeline (green) and

BASALT (red) from human gut Dataset 2, chicken gut, hot spring, and AD samples. There were nearly no difference of N50 values between shared MAGs retrieved by BASALT and MAG-HiFi pipeline. The boxplot shows the distribution of data, the central dot in the box represents the median, the box bounds represent the 25th and 75th percentiles, and whiskers represent the minima to maxima values.

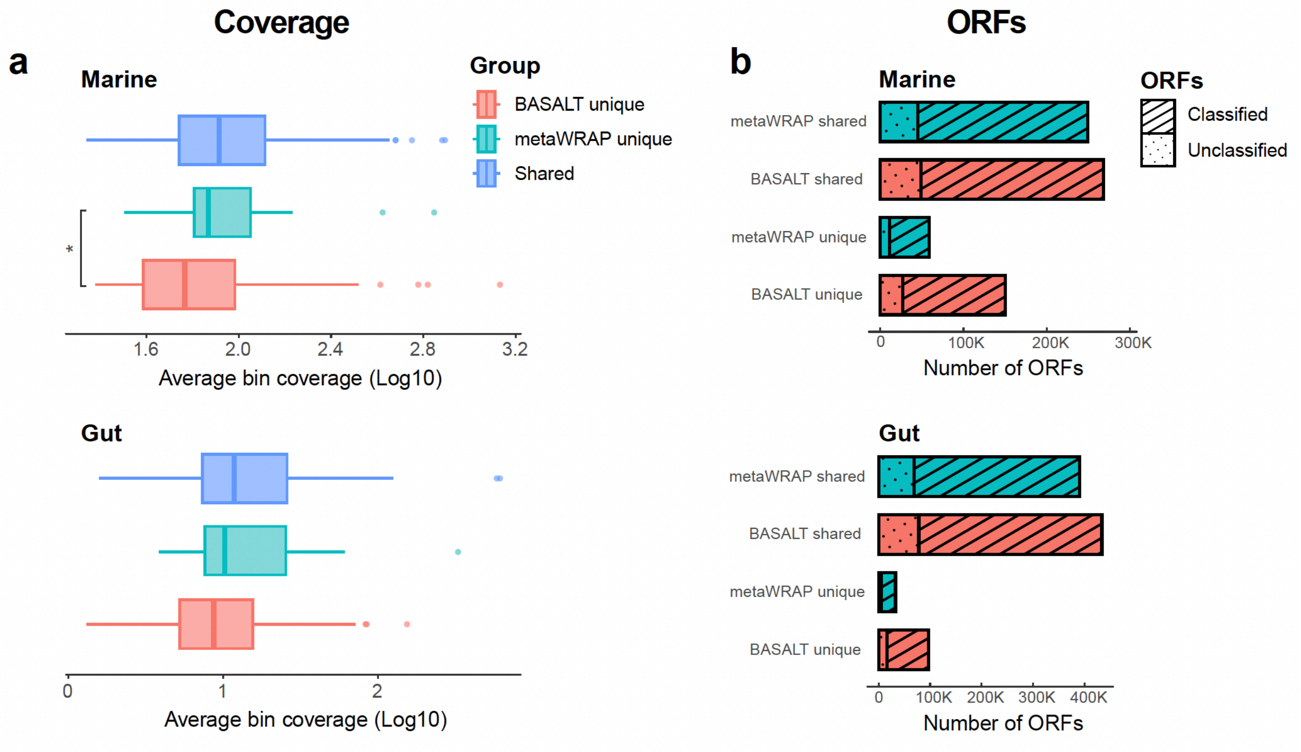

Supplementary Figure 9. Comparison of MAGs obtained by BASALT vs. metaWRAP from marine (n = 77) and human gut Dataset 1 (n = 140) samples. a) Boxplot of average bin coverage. Significant differences between MAGs unique to BASALT (red) and MAGs unique to metaWRAP (cyan) were determined by $P < 0.05$ in marine dataset (Kruskal-Wallis test, $P = 0.015$), while no significant difference was found in human gut dataset (Kruskal-Wallis test, $P = 0.13$). The boxplot shows the distribution of data, the central dot in the box represents the median, the box bounds represent the 25th and 75th percentiles, and whiskers represent the minima to maxima values. b) Summary of ORFs predicted in MAGs obtained by BASALT (red) or metaWRAP (cyan) from marine and human gut datasets. There were more classified and unclassified ORFs found in BASALT MAGs than metaWRAP MAGs in both unique and shared MAGs.

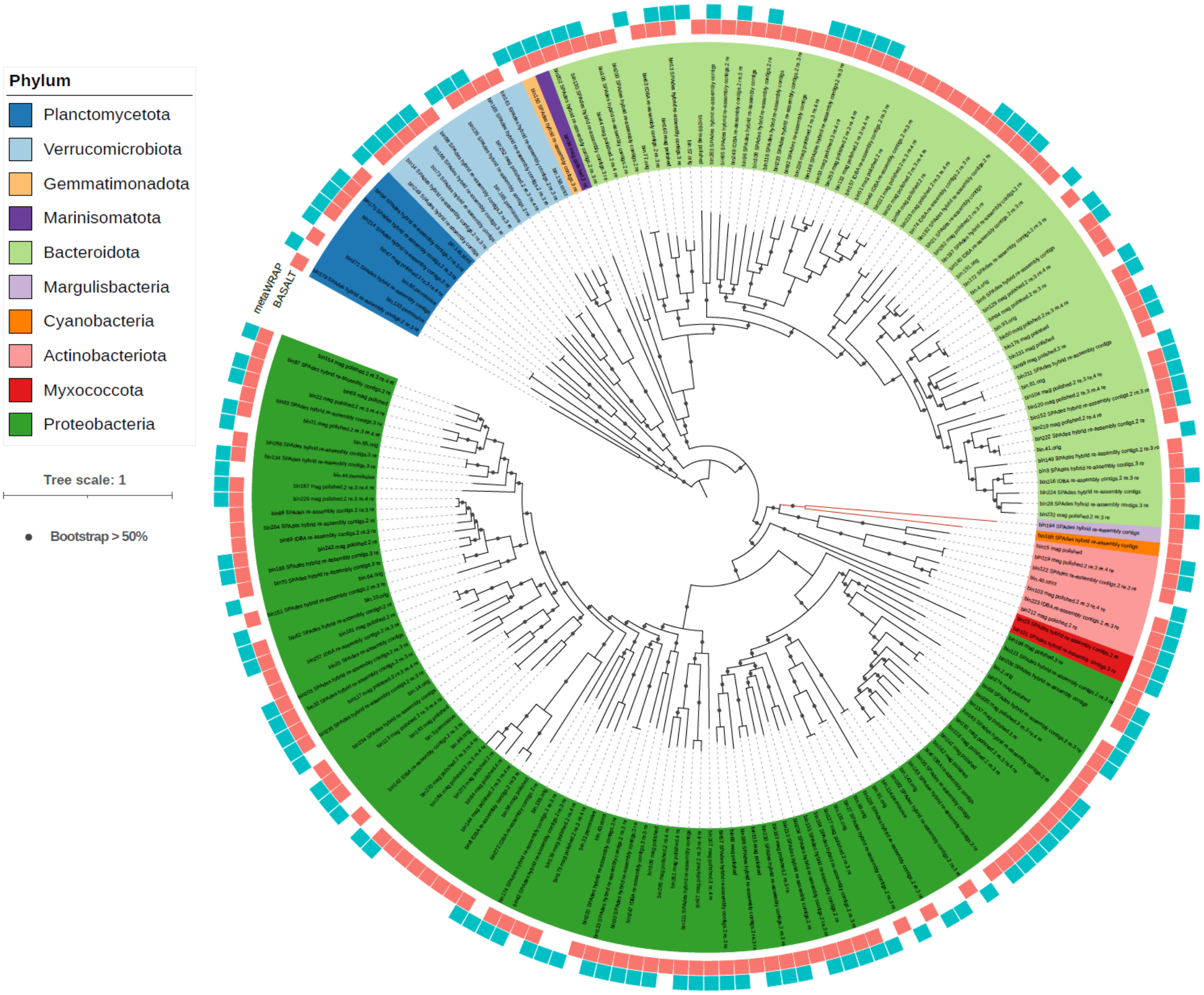

Supplementary Figure 10. Phylogenetic tree of bacterial MAGs recovered from marine samples based on 120 concatenated marker genes. Mid-point rooted phylogenetic tree was constructed using IQ-TREE with 1,000 bootstraps and best fit models LG+F+R9. Blocks in the outer circles indicate corresponding MAGs recovered by BASALT (red) or metaWRAP (cyan). Black dots in the middle of branches indicate >50% bootstrap support, and branches highlighted in red indicate unique lineages at phylum level obtained by BASALT.

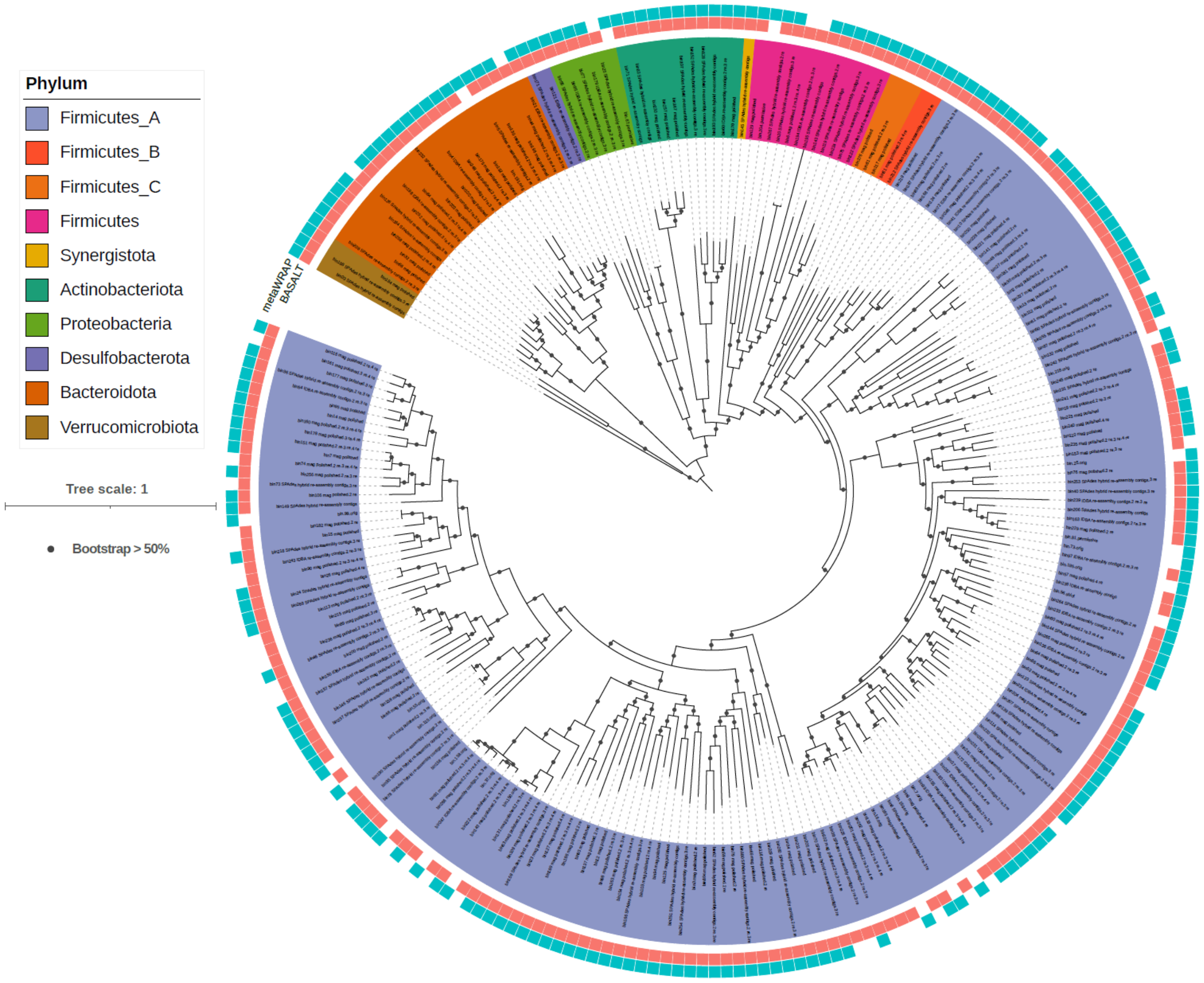

Supplementary Figure 11. Phylogenetic tree of bacterial MAGs recovered from human gut samples based on 120 concatenated marker genes. Mid-point rooted phylogenetic tree was constructed using IQ-TREE with 1,000 bootstraps and best fit models LG+ R8. Blocks in the outer circles indicate corresponding MAGs recovered by BASALT (red) or metaWRAP (cyan). Black dots in the middle of branches indicate >50% bootstrap support.

Supplementary References

1. Stewart, R.D. et al. Compendium of 4,941 rumen metagenome-assembled genomes for rumen microbiome biology and enzyme discovery. *Nat. Biotechnol.* **37**, 953-961 (2019).
2. Ioffe, S. & Szegedy, C. in International conference on machine learning 448-456 (pmlr, 2015).
3. He, K., Zhang, X., Ren, S. & Sun, J. in Proceedings of the IEEE conference on computer vision and pattern recognition 770-778 (2016).
4. Paszke, A. et al. Pytorch: An imperative style, high-performance deep learning library. *Advances in neural information processing systems* **32** (2019).
5. Loshchilov, I. & Hutter, F. Decoupled weight decay regularization. *arXiv preprint arXiv:1711.05101* (2017).
