# Supplementary material for: BASALT refines binning from metagenomic data and increases resolution of genome-resolved metagenomic analysis
Source: Nat Commun. 2024 Mar 11;15:2179. doi: 10.1038/s41467-024-46539-7 (PMC10928208; doi:10.1038/s41467-024-46539-7)
Supplement: Supplementary file 3 — Description of Additional Supplementary Files [file 41467_2024_46539_MOESM3_ESM.pdf]

Description of Additional Supplementary Files

File name: Supplementary Data 1

Description: Bin stats of CAMI-high datasets using BASALT after Bin Selection, Refinement, and Gap Filling Modules

File name: Supplementary Data 2

Description: Bin stats of CAMI-high datasets using BASALT (B), metaWRAP (M), DASTool (D), and VAMB (V), respectively. B = BASALT, M = metaWRAP, D = DASTool, V = VAMB.

File name: Supplementary Data 3

Description: Summary of bins recovered from CAMI-medium and CAMI-high datasets using BASALT, DASTool, metaWRAP and VAMB based on SPAdes or MEGAHIT assemblies. CA = Co-assembly, SA = Single assembly, CAB = Co-assembly and refined with BASALT, CPN = Completeness, CTN = Contamination

File name: Supplementary Data 4

Description: Average coverage of MAGs obtained from Aiding Lake sediment samples using BASALT or metaWRAP

File name: Supplementary Data 5

Description: Summary of BASALT unique modules

File name: Supplementary Data 6

Description: Taxonomic classification against GTDB of MAGs obtained from Aiding Lake sediment samples using BASALT or metaWRAP. Unique lineages at class (bacteria) or order (archaea) level were highlighted with red.

File name: Supplementary Data 7

Description: List of available binning tools integrated in BASALT

File name: Supplementary Data 8

Description: Information of unique lineages obtained by BASALT

File name: Supplementary Data 9

Description: Details of downloaded datasets

File name: Supplementary Data 10

Description: Summary of MAGs obtained from Aiding Lake sediment samples, human gut samples, and marine samples, respectively. HQ MAGs = high-quality MAGs (completeness >= 90, contamination <= 5), MQ MAGs = medium-quality MAGs (completeness >= 50, contamination <= 10).
