# Supplementary material for: BASALT refines binning from metagenomic data and increases resolution of genome-resolved metagenomic analysis
Source: Nat Commun. 2024 Mar 11;15:2179. doi: 10.1038/s41467-024-46539-7 (PMC10928208; doi:10.1038/s41467-024-46539-7)
Supplement: Supplementary file 5 — Reporting Summary [file 41467_2024_46539_MOESM5_ESM.pdf]

nature portfolio

Corresponding author(s): Ke Yu

Last updated by author(s): Feb 15, 2024

Reporting Summary

Nature Portfolio wishes to improve the reproducibility of the work that we publish. This form provides structure for consistency and transparency in reporting. For further information on Nature Portfolio policies, see our Editorial Policies and the Editorial Policy Checklist.

Statistics

For all statistical analyses, confirm that the following items are present in the figure legend, table legend, main text, or Methods section.

n/a	Confirmed	
☐	☒	The exact sample size (*n*) for each experimental group/condition, given as a discrete number and unit of measurement
☐	☒	A statement on whether measurements were taken from distinct samples or whether the same sample was measured repeatedly
☐	☒	The statistical test(s) used AND whether they are one- or two-sided<br>*Only common tests should be described solely by name; describe more complex techniques in the Methods section.*
☒	☐	A description of all covariates tested
☒	☐	A description of any assumptions or corrections, such as tests of normality and adjustment for multiple comparisons
☐	☒	A full description of the statistical parameters including central tendency (e.g. means) or other basic estimates (e.g. regression coefficient) AND variation (e.g. standard deviation) or associated estimates of uncertainty (e.g. confidence intervals)
☐	☒	For null hypothesis testing, the test statistic (e.g. *F*, *t*, *r*) with confidence intervals, effect sizes, degrees of freedom and *P* value noted<br>*Give P values as exact values whenever suitable.*
☒	☐	For Bayesian analysis, information on the choice of priors and Markov chain Monte Carlo settings
☒	☐	For hierarchical and complex designs, identification of the appropriate level for tests and full reporting of outcomes
☒	☐	Estimates of effect sizes (e.g. Cohen's *d*, Pearson's *r*), indicating how they were calculated

Our web collection on statistics for biologists contains articles on many of the points above.

Software and code

Policy information about availability of computer code

Data collection	Datasets from NCBI BioProject were downloaded using fastq-dump (v2.9.6). Simulated long-read sequences of CAMI-high datasets were generated using CAMISIM (v1.3). All other data were downloaded manually.
Data analysis	The following softwares were used in this study:<br>SPAdes (v3.14.1), MEGAHIT (v1.2.9), IDBA-UD (v1.1.1), MetaBAT2 (v2.12.1), MaxBin2 (v2.2.4), CONCOCT (v1.1.0), VAMB (v3.0.2), DASTool (v1.1.2), metaWRAP (v1.3.2), Bowtie2 (v2.4.2), BWA (v0.7.17-r1188), SAMtools (v1.7), CheckM (v1.1.3), BLAST (v2.11.0+), Diamond (v2.0.6.144), Pilon (v1.23), Prodigal (v2.6.3), HMMER (v3.1b2), GTDB-Tk (v1.5.0), IQ-TREE (v2.1.3), dRep (v2.5.4), CheckM2 (v1.0.1), SemiBin2 (v1.5.1). All BASALT codes, including in-house scripts for quality checking against benchmarking datasets, are available at Github (https://github.com/EMBL-PKU/BASALT) and Zenodo (https://doi.org/10.5281/zenodo.10653187).

For manuscripts utilizing custom algorithms or software that are central to the research but not yet described in published literature, software must be made available to editors and reviewers. We strongly encourage code deposition in a community repository (e.g. GitHub). See the Nature Portfolio guidelines for submitting code & software for further information.

Data

Policy information about availability of data

All manuscripts must include a data availability statement. This statement should provide the following information, where applicable:

- Accession codes, unique identifiers, or web links for publicly available datasets
- A description of any restrictions on data availability
- For clinical datasets or third party data, please ensure that the statement adheres to our policy

The CAMI benchmarking datasets were downloaded at CAMI Challenge (https://data.cami-challenge.org/participate). All tested genomes are available in the CNCB-NGDC database (https://ngdc.cncb.ac.cn/) under projects PRJCA014711 (https://ngdc.cncb.ac.cn/search/all?q=PRJCA014711). Metagenomic sequence data of Aiding Lake are available in the CNCB-NGDC database under projects PRJCA014712 (https://ngdc.cncb.ac.cn/search/all?q=PRJCA014712). Real datasets were downloaded from:
ENA sequence archive: PRJEB52999 (https://www.ebi.ac.uk/ena/browser/view/PRJEB52999), PRJEB48021(https://www.ebi.ac.uk/ena/browser/view/PRJEB48021)
NCBI sequence archive: PRJNA820119 (https://www.ncbi.nlm.nih.gov/bioproject/?term=PRJNA820119), PRJNA648801 (https://www.ncbi.nlm.nih.gov/bioproject/?term=PRJNA648801), PRJNA681475 (https://www.ncbi.nlm.nih.gov/bioproject/?term=PRJNA681475), PRJNA750084 (https://www.ncbi.nlm.nih.gov/bioproject/?term=PRJNA750084), PRJNA595610 (https://www.ncbi.nlm.nih.gov/bioproject/?term=PRJNA595610), PRJNA748109 (https://www.ncbi.nlm.nih.gov/bioproject/?term=PRJNA748109)
DDBJ sequence archive: DRR290133 (https://ddbj.nig.ac.jp/resource/sra-run/DRR290133)

Research involving human participants, their data, or biological material

Policy information about studies with human participants or human data. See also policy information about sex, gender (identity/presentation), and sexual orientation and race, ethnicity and racism.

Reporting on sex and gender	N/A
Reporting on race, ethnicity, or other socially relevant groupings	N/A
Population characteristics	N/A
Recruitment	N/A
Ethics oversight	N/A

Note that full information on the approval of the study protocol must also be provided in the manuscript.

Field-specific reporting

Please select the one below that is the best fit for your research. If you are not sure, read the appropriate sections before making your selection.

☒ Life sciences ☐ Behavioural & social sciences ☐ Ecological, evolutionary & environmental sciences

For a reference copy of the document with all sections, see nature.com/documents/nr-reporting-summary-flat.pdf

Life sciences study design

All studies must disclose on these points even when the disclosure is negative.

Sample size	For assessment of BASALT performance and benchmarking comparison, this study used CAMI-high and CAMI-medium benchmarking datasets that comprise 596 and 132 genomes, respectively (Sczyrba, A. et al. Critical assessment of metagenome interpretation—a benchmark of metagenomics software. Nat. Methods 14, 1063-1071 (2017)); For case-study of Aiding Lake, 4 lake sediment samples (n=4) of biologically replicates were collected. This sample size is designed to balance the coverage of Aiding Lake sediment microbiome, ensure the depth of sequencing data, and maintain the computational capacity given the available resources.
Data exclusions	No data was excluded in this study.
Replication	Analysis of Aiding Lake sediment samples was conducted with replicated samples (n=4) in representative of Aiding Lake sediment microbiome. All attempts at replication were successful.
Randomization	Lake sediment samples were randomly collected at 0-10 cm depth in the lake, with 50 m apart between each two samples. No randomization was performed as limited number of samples were collected.
Blinding	Blinding is not applicable to this study because it was necessary to track each sample with ids during the metagenomic analysis.

Reporting for specific materials, systems and methods

We require information from authors about some types of materials, experimental systems and methods used in many studies. Here, indicate whether each material, system or method listed is relevant to your study. If you are not sure if a list item applies to your research, read the appropriate section before selecting a response.

Materials & experimental systems

n/a	Involved in the study
☒	☐ Antibodies
☒	☐ Eukaryotic cell lines
☒	☐ Palaeontology and archaeology
☒	☐ Animals and other organisms
☒	☐ Clinical data
☒	☐ Dual use research of concern
☒	☐ Plants

Methods

n/a	Involved in the study
☒	☐ ChIP-seq
☒	☐ Flow cytometry
☒	☐ MRI-based neuroimaging

Plants

Seed stocks	N/A
Novel plant genotypes	N/A
Authentication	N/A
